# The Complex Metabolomics Crosstalk Triggered by Four Molecular Elicitors in Tomato

**DOI:** 10.3390/plants11050678

**Published:** 2022-03-01

**Authors:** Giusy Iula, Begoña Miras-Moreno, Youssef Rouphael, Luigi Lucini, Marco Trevisan

**Affiliations:** 1 Department for Sustainable Food Process, Università Cattolica del Sacro Cuore, 29122 Piacenza, Italy; giusy.iula@unicatt.it (G.I.); luigi.lucini@unicatt.it (L.L.); marco.trevisan@unicatt.it (M.T.); 2 Department of Agriculture, University of Naples Federico II, 80055 Naples, Italy

**Keywords:** *Solanum lycopersicum* L., metabolomics, phenylpropanoids, chitosan, polyamines, sustainable agriculture, phytohormones, biotic and abiotic stressors

## Abstract

The elicitation of plant secondary metabolism may offer interesting opportunities in the framework of sustainable approaches in plant science and in terms of their ability to prime resistance to biotic and abiotic stressors. The broad metabolic reprogramming triggered by different molecular elicitors, namely salicylate (SA), polyamines (PAs), and chitosan, was comprehensively investigated using a metabolomics approach and the tomato (*Solanum lycopersicum* L.) as the model crop. Six different treatments were compared: a negative control (no treatments), a second negative control treated with 1 M acetic acid (the reference for chitosan, since chitosan was solubilized in acetic acid), and four molecular elicitors, 1 mM 2,1,3-benzothiadiazole (BTH, a positive control), 10 mg/mL chitosan, 0.01 mM SA, and a 0.1 mM PA (putrescine, spermidine, and spermine). All treatments determined a slight increase in biomass, in particular following PA treatment. A broad reprogramming of secondary metabolism could be observed, including membrane lipid remodeling, phenylpropanoid antioxidants, and phytohormone crosstalk. Overall, our results suggest that PAs, SA, and BTH shared a systemic acquired resistance (SAR)-related response, whereas chitosan induced a more distinct induced systemic resistance (ISR)-like jasmonate-related response. These results pave the way towards the possible use of elicitors as a sustainable tool in plant science and agriculture by increasing crop resilience to biotic and abiotic stressors without detrimental effects on plant biomass.

## 1. Introduction

Plants are subjected to different sources of abiotic and biotic stresses, such as salinity, floods, drought, extremes in temperature, and heavy metals. Examples of biotic stress include the attacks by pathogens, such as fungi, bacteria, and nematodes, as well as herbivores [1]. Consequently, plants have evolved different defense strategies to deal with stress factors [2], including the so-called priming system [3]. Priming is a mechanism leading to a physiological state that enables plants to respond more rapidly and robustly to stress. The primed state is not a mere activation of plant defense responses, but rather an improved perception and/or amplification of defense [4]. Until now, it is not truly clear the way in which the priming acts in the cells [5]. It has been proposed that priming involves the accumulation of inactive cellular proteins playing a role in cellular signal amplification [6] in a way that they are ready to activate their defenses faster and more efficiently when a stress agent acts on plants [7].

The plant immune response comprised the systemic acquired resistance (SAR) and induced systemic resistance (ISR). SAR is a form of induced resistance that can be activated upon pathogen infection, but it can be also induced by exogenous application of molecular elicitors [8]. SAR is characterized by a local and systemic increase in salicylic acid (SA) levels [9] and by upregulation of a set of SAR genes producing the pathogenesis-related (PR) proteins. These PR proteins possess antimicrobial activity and are thought to contribute to the state of resistance attained [9]. However, induced systemic resistance does not rely on the presence of an invading pathogen, is triggered by jasmonates, and activates physical or chemical barriers in the host plant [10].

Since it has been demonstrated that some elicitors are able to activate the SAR/ISR [8], they become more attractive to be used as priming agents. The first use of the priming strategy refers to seed treatment in pre-sowing, a process that enables seeds to germinate more efficiently [11]. Since this treatment has been demonstrated to be efficient in modulating plant secondary metabolism, this paves the way towards the possibility of priming the plant itself at later growth stages. Different stimuli can be used to trigger secondary metabolism, and these so-called elicitors can be both biotic and abiotic. Among abiotic factors, benzothiadiazole (BTH) has a known effect on induction of the primed state, representing a functional analogue of the plant endogenous hormone, SA [12]. In literature, it has been demonstrated that BTH is a potent inductor of pathogenesis-related genes, priming the plants for potential pathogenic attacks [13,14]. Similarly, SA itself, a naturally occurring plant hormone, has been used as a priming agent [15]. SA modulates many cellular processes during abiotic stresses [16] and is involved in processes, such as stomatal closure, ion uptake and transport, ethylene biosynthesis, transpiration, stress tolerance, membrane permeability, photosynthesis, and growth [17]. Another elicitor molecule used for priming is chitosan, a natural linear polysaccharide [18] that composes part of the carbohydrate skeleton of fungal cell walls [19]. This molecule elicits microbe-associated molecular patterns (MAMPs) in plant by pattern recognition receptors (PRRs) [20], thus leading to the activation of plant immunity. Therefore, chitosan can induce systemic acquired immunity [21]. Another important and less studied class of organic molecules that is supposed to be effective in priming induction is represented by polyamines (PAs) [22]. The most common PAs in plants are putrescine (Put), spermidine (Spd), and spermine (Spm) [23]. They play important roles in plant growth, developmental processes, and environmental stress responses. Particularly, they have a complex role in oxidative stress as they can increase the activity of antioxidant enzymes in plants, therefore, protecting them from environmental factors [24]. Moreover, it has also been demonstrated that spermidine has a positive role in effectively suppressing the multiplication of the cucumber mosaic virus in *Arabidopsis* [25]. Therefore, due to these positive stress response roles, they have been proposed as elicitors of the primed state [24].

To date, most of the previous work on priming agents has been focused on defense-related molecular patterns, even though it can be postulated that these molecules may trigger a wider series of metabolic processes given their ability to prompt signaling cascade responses. A broader comprehension of the processes modulated by elicitors can provide insights into the complex plant responses to priming, thus, paving the way towards sustainable solutions in plant defense. On this basis, our work aimed to unravel the metabolic processes underlying priming by non-biotic elicitors in a secondary metabolism framework that includes, but is not limited to, defense-related mechanisms. Towards this objective, the tomato (*Solanum lycopersicum* L.) has been chosen as the model because of its relevance worldwide [26,27,28] and because of its diverse secondary metabolism. To achieve the goal, metabolomics were used due to their comprehensive nature, which is suitable to investigating the primed state in tomato plants in a hypothesis-free, untargeted manner.

## 2. Results

### 2.1. Biomass Production

Biometric parameters in response to elicitors were recorded after 15 days of treatment and are reported in Table 1 and shown in Supplementary Figure S1. The application of elicitors resulted in a significant increase of fresh biomass production in all the cases but chitosan compared to the control, while no significant differences were found regarding dry weight. In particular, the application of PAs had the greatest effect on fresh biomass, reaching 1.5-fold the biomass in control.

### 2.2. Effects of Treatments on Tomato Leaves Metabolic Profiles

An untargeted metabolomics approach was used to comprehensively investigate the biochemical processes affected by the different elicitors in tomato leaves. Overall, more than 3000 putative metabolites were annotated using the database, PlantCyc 12.6. The whole list of annotated compounds, together with individual abundances and composite mass spectra (mass and abundance combinations), are listed in Supplementary Table S1.

**Figure 1:**
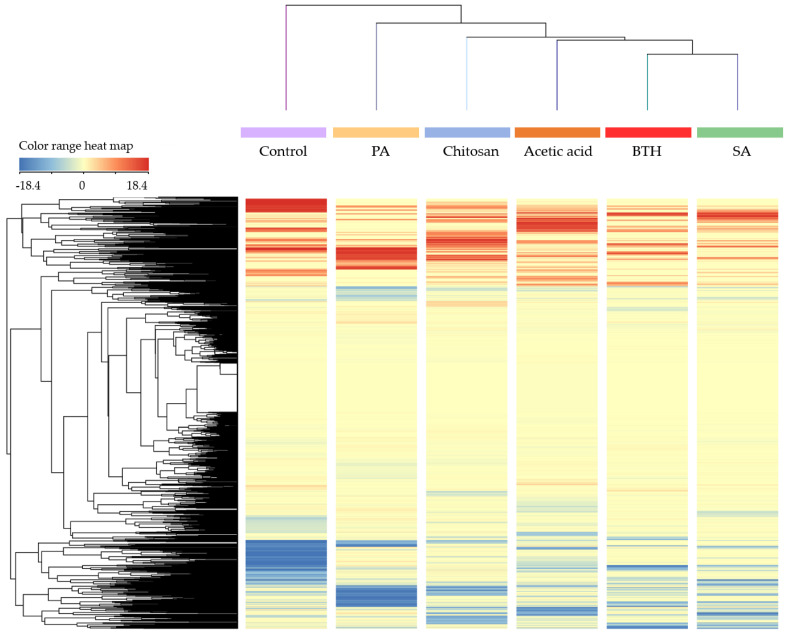
Unsupervised hierarchical cluster analysis of the whole metabolomics profiles in tomato leaves following the different elicitor treatments. Metabolites were obtained through UHPLC-ESI/QTOF-MS untargeted analysis, and their intensities were used to create the heat map in the figure, based on fold-change values (color range heat map is provided on top left). The cluster on top refers to similarities across treatments, whereas the cluster on left reports compound clusters (both according to Euclidean distance and Ward’s linkage rule).

Multivariate statistics were then used to investigate relatedness/unrelatedness between treatments. The unsupervised hierarchical cluster analysis (HCA) indicated that leaf metabolic profile was significantly and distinctively influenced by the addition of the elicitors (Figure 1). In more details, two principal clusters were observed, separating the control from the treated plants. Among treated plants, SA clustered closer to BTH and presented the most distinct profile compared to the control.

**Figure 2:**
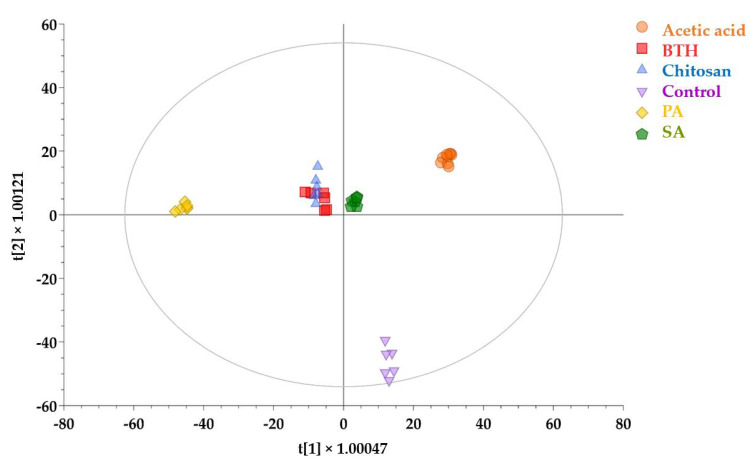
Supervised discrimination of the metabolomics profile in tomato leaves following the treatment with different elicitors. The score plot in figure was produced by orthogonal projection to latent structures discriminant analysis (OPLS-DA) modeling according to first and second latent vectors, t[1] and t[2], respectively. The model correlation with the dataset was R^2^Y = 0.99, whereas prediction ability was Q^2^Y = 0.92.

Afterwards, a supervised approach based on orthogonal projection to latent structures discriminant analysis (OPLS-DA) allowed us to separate the samples in the score plot space according to the treatments. This supervised modeling allowed for confirming the presence of distinct metabolic profiles in treated leaves compared to the control (Figure 2) and in agreement with HCA. In fact, samples corresponding to differently treated plants were distributed along the first latent vector, while the second component clearly separated the control samples from the treated. These multivariate modelings also revealed that chitosan, BTH, and SA had closer metabolic profiles, while PAs showed the most distinguished profile. The variable importance in projection (VIP) analysis following OPLS-DA identified 100 metabolites with the highest discriminant potential (VIP score > 1.25). The most represented biochemical classes of compounds belonged to hormones, phenylpropanoids, and nitrogen-containing secondary metabolites (Supplementary Table S2).

**Figure 3:**
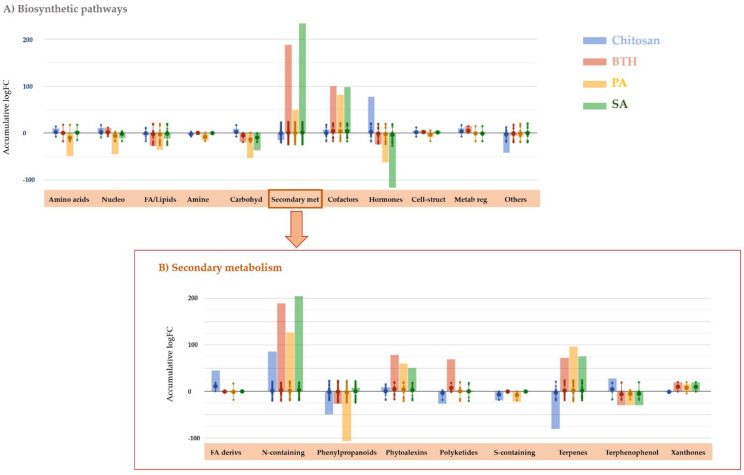
Metabolic processes (**A**) and secondary metabolite biosynthesis (**B**) modulated in tomato leaves following the treatments with elicitors. The metabolomics dataset produced through UHPLC-ESI/QTOF-MS was subjected to ANOVA and FC analysis (*p* < 0.01, FC ≥ 7), and differential metabolites were loaded into the PlantCyc Pathway Tool (https://www.plantcyc.org/ Plant Metabolic Network; accessed on 20 November 2021). The x axis represents each set of metabolic subcategories, while the y axis corresponds to the accumulative log fold change (FC). The large dots represent the average (mean) of all FCs for the different metabolites in the class, while the small dots represent the individual log FC. The abbreviated subcategory names on the x axis correspond to: Nucleo: nucleosides and nucleotides; FA/lipids: fatty acids and lipids; Amines: amines and polyamines; Carbohyd: carbohydrates; Secondary met: secondary metabolism; Cofactors: cofactors, prosthetic groups, electron carriers, and vitamins; Cell-struct: plant cell structures; Metab reg: metabolic regulators; FA derivs: fatty acid derivatives; N-containing: nitrogen-containing secondary metabolism; S-containing: sulfur-containing secondary metabolism; Terpenophenol: terphenophenolic compounds.

Once the distinguish effect of each elicitor on tomato leaves was confirmed, the specific molecular processes involved in plant response were investigated. With this purpose, the discriminant compounds derived from the ANOVA and FC analysis (Supplementary Table S3) were interpreted using the Omic Viewer Pathway Tool according to their biosynthetic pathway (Figure 3).

The most remarkable result was the strong modulation of secondary metabolism. Notably, PA-, BTH-, and SA-treated plants showed an accumulation of secondary metabolites, while chitosan provoked a general decrease of these compounds (Figure 3A). Despite this general trend, leaves accumulated N-containing secondary metabolites after applying elicitors, including chitosan. Among others, the nitrogen-containing glucosinolates (23 compounds) and alkaloids (42 compounds), together with phenylpropanoids and terpenes (77 and 43 compounds, respectively) were the secondary metabolites showing the most extensive modulation following the treatment with elicitors. These elaborations confirmed a broad and diverse modulation of secondary metabolism, rather than a more specific or limited response to treatments. A detail of these four main classes of secondary metabolites is provided as Supplementary Figure S1, where individual fold-change values are plotted for each treatment. Among these compounds, alkaloid accumulation was positively correlated with all the treatment while glucosinolates were increased solely by chitosan and, to a lesser extent, in the presence of SA. Strong accumulation was observed for terpenes (except for chitosan, which provokes a decrease of isoprenoid-related compounds). In the case of phenylpropanoids, chitosan, BTH, and PAs decreased several compounds of this class, while SA elicited them. The specific phenolic compounds impacted by the treatments seemed to match for SA, PAs, and BTH, while chitosan modulated other specific compounds within this class. Arogenate, the precursor of Phe and Tyr and, therefore, the intermediate of several secondary metabolites, including phenylpropanoids, was strongly accumulated in the presence of chitosan and strongly repressed in the presence of PAs, BTH, and SA. A similar trend was found for Tyr. Concerning plant defense, all treatments triggered an accumulation of phytoalexins and polyketides.

Fatty acids, sterols, phospholipids, and glycolipids involved in these biosynthetic pathways were affected by the elicitors. Interestingly, the accumulation of sterols was promoted by all treatments, while glycolipids decreased in all the cases. Moreover, phosphatidylcholines (i.e., 1-linoleoyl-2-oleoyl-phosphatidylcholine, 1-linoleoyl-2-α-linolenoyl-phosphatidylcholine, and 1-palmitoyl-2-oleoyl-phosphatidylcholine) were positively modulated in the case of BTH, SA, and, to a lesser extent, PAs. Furthermore, 1-oleoyl-2-(3E)-hexadecenoyl-phosphatidylglycerol increased in the sole presence of chitosan, whereas monogalactosyldiacylglycerols (MGDG) decreased in plants treated with BTH, PAs, and SA and, to a lesser extent, with chitosan.

On the other hand, a modulation of phytohormones was observed after applying elicitors. Similar to secondary metabolism, chitosan induced a more distinctive pattern regarding phytohormones, while BTH, PAs, and SA elicited more comparable trends. In detail, auxins and brassinosteroids decreased in the presence of BTH, PAs, and SA, while they increased in the presence of chitosan. Abscisic aldehyde strongly increased after applying BTH, PAs, and SA, while abscisic alcohol decreased in all the cases. Cytokinins were strongly modulated, and this modulation seemed to be treatment-dependent. Benzoates involved in the SA biosynthetic pathway accumulated in the presence of BTH, SA, and PAs. Interestingly, the jasmonic acid (JA)-signaling cascade was also involved in the response to elicitors. In this sense, (−)-jasmonoyl-L-isoleucine and (+)-7-epi-jasmonate accumulated in chitosan-treated plants. BTH and SA accumulated (−)-jasmonoyl-1-aminocyclopropane-1 carboxylate and (+)-7-epi-jasmonoyl-L-isoleucine, while they were repressed in the presence of PAs.

Finally, vitamin E biosynthetic pathways were notably stimulated after adding all treatments but chitosan. In fact, homogentisate, 2-methyl-6-phytyl-1,4-benzoquinol, and the final product tocotrienol strongly accumulated in PA-, BTH-, and SA-treated plants.

## 3. Discussion

It is well-known that elicitors can be used for increasing plant defense and plant resistance, even in the absence of the stress, since plants can perceive elicitors as signal-inducing compounds and respond to them [29]. In some cases, elicitors can lead to the activation of the SAR and ISR plant defense pathways [30]. However, there is a lack of knowledge about the specific molecular reprogramming triggered by the elicitors, knowledge that is essential for an appropriate application of these molecules in the framework of sustainable agriculture.

In our work, we investigated the effect of some elicitors, namely BTH (as a positive control), chitosan, SA, and PAs on tomato leaves, to unravel the molecular mechanisms that take place after their spraying. Our results confirmed the ability of these elicitors for triggering plant biochemical responses, in agreement with previous results [29]. N-containing secondary metabolites, isoprenoids, and phenylpropanoids were strongly modulated by the elicitors. Moreover, phytoalexins, plant defense metabolites, accumulated in response to the treatments. It is noteworthy that many of the biosynthetic pathways involved in plant response seemed to be modulated in a treatment-dependent manner suggesting a specific response to elicitors. It was clear that hormones (including JA, ethylene, and abscisic acid (ABA)) are involved in the signaling cascade that takes place after the elicitor application. However, the plant defense response implies a complex network and crosstalk responses that can be positively and negatively interconnected [4]. In our work, SA, JA, and ABA derivatives were found as discriminant compounds following elicitation compared to the control, indicating a strong involvement of hormonal imbalance in plant response to our external stimuli. This intricate cross-communicating hormone signaling, involving several plant regulators, is common in plant immunity and may imply an advantage to rapidly adapting to biotic and abiotic stresses [31].

Despite all the elicitors tested in this work presented specific effects on tomato leaves, two general mechanisms seemed to occur across treatments. On the one hand, PAs and BTH seemed to involve the same pathways as SA, suggesting the implication of the SA signal cascade, which is supported by the biosynthesis of benzoates and its derivatives. Despite some differences, the elicitation with PAs, BTH, and SA led to the accumulation of the same classes of secondary metabolites that were not observed for chitosan, with the latter triggering a different biochemical reprogramming.

BTH is a synthetic SA analog. The application of both SA and BTH involved the lipid biosynthetic pathway, including phospholipids. Interestingly, some evidence connecting phospholipid signaling and SA-dependent immunity agree with our results. However, although SA and BTH seemed to strongly modulate lipid biosynthesis, PAs also modulated this pathway. In this sense, it is known that PAs also play an important role in plant signaling and have been correlated to the SA signaling cascade [32]. Lipid-mediated signalling is involved in plant adaptation to environmental stresses and can mitigate stressors probably by modulating membrane properties. Processes such as wax and cutin deposition, rather than suberization, have been linked to the anti-stress function of lipid remodelling [33]. Moreover, phospholipid accumulation under stress conditions is associated with the activation of phospholipases (PL) and membrane modifications that occur during defense [34]. As previously mentioned, lipids (including phosphatidic acid) play an important role in plant signaling in response to PAs, BTH, and SA. Phosphatidic acids are signaling molecules that can be modulated by several stimuli. Phosphatidic acids regulate cellular processes, including lipid metabolism, signal transduction, cytoskeletal rearrangements, and vesicular trafficking, and seem to induce ROS production by activating NADPH [35].

In addition, MGDG, essential chloroplast membrane lipids, were strongly downregulated in the presence of PAs, SA, and BTH. Previous studies revealed that the alteration in membrane lipids and the reduction of MGDG is a common adaptation plant strategy to several abiotic stresses to maintain the chloroplast structure under adverse conditions by promoting proper stacking and development of thylakoid membranes and proteins [36].

Considering that chloroplast lipids play an important role in plant tolerance, this could explain the differences between PA, BTH, and SA, and chitosan regarding the biomass. Another class of lipids involved in plant signalling is oxylipins. The phytohormone, JA, is the best-known oxylipin involved in the activation of various defense responses [36]. In fact, JAs were also modulated suggesting a complex interaction between SA signalling and other plant regulators after the elicitor addition.

Concerning lipids and lipid peroxidation, the biosynthetic pathway of vitamin E (tocopherols and tocotrienols) was strongly stimulated in the presence of PAs, BTH, and SA. Synthesized from homogentisic acid and isopentenyl diphosphate in the plastid envelope, tocopherols and tocotrienols are essential to maintain membrane integrity by scavenging lipid peroxyl radicals and to prevent the propagation of lipid peroxidation in membranes. Interestingly, some authors have found a positive correlation between SA and the biosynthesis of tocopherol under stress conditions [37]. Moreover, α-tocopherol could also act on the diacylglycerol pathway by activating diacylglycerol kinase, and consequently decreasing diacylglycerol and protein kinase C activation, which is in agreement with our results [38].

Unlike PAs, SA, and BTH that are also endogenous elicitors, chitosan arises from cell wall polysaccharides that can be recognized by plants and that can activate PAMP. After being recognized by plant PRRs, chitosan triggers plant defense responses inducing non-host resistance and priming systemic immunity. The mechanism proposed for chitosan perception involved the increase in H^+^ and Ca^2+^ influx into the cytosol, the activation of MAP-kinases, and the synthesis of ABA, JAs, phytoalexins, among others [39]. Despite the modulation of secondary metabolism, our findings revealed a strong effect of chitosan on plant hormones, as previously reported by several authors [40]. In this line, our results agree with the previous studies since JA signaling was involved in the plant response to chitosan rather than SA-mediated signaling. In fact, it has been reported that the application of chitosan, chitinase, and glucanase activities led to a rapid increase JA content, activating the octadecanoic pathway. This pathway includes the biosynthesis of JAs following the oxidation of linolenic acid and led to accumulating phytoalexins [39]. Our findings confirm the implication of JAs in chitosan elicitation and the subsequent accumulation of phytoalexins and other secondary metabolites. Among others, glucosinolates, which are modulated by JAs, accumulated in response to chitosan but not for the other elicitors. Other plant defense-related compounds increasing after chitosan application included anthocyanins glucosides, which are important ROS scavengers [41]. In fact, although all elicitors modulated phenylpropanoid biosynthesis, the specific phenolic classes being modulated were similar for BTH, SA, and PAs and diverse for chitosan. Phe is regulated by the key enzyme, arogenate dehydratases (ADTs), catalyzing the conversion of arogenate into Phe and representing one of the principal routes to produce Phe together with phenylpiruvate. In our study, arogenate and Tyr were strongly accumulated in the presence of chitosan but not in the presence of the other elicitors. In fact, it has been reported that chitosan modulates PAL in many hosts [39]. p-Hydroxyphenylpyruvate (HPP), the first intermediate in tocopherol biosynthesis, is formed from prephenate via arogenate and Tyr. In most plants, arogenate is transformed into Tyr by arogenate dehydrogenase and into Phe by arogenate dehydratase that is, in turn, strongly feedback inhibited by Phe. Tyrosine aminotransferase, a key enzyme in the biosynthesis of tocopherols, catalyzes the formation of HPP from tyrosine in plants. Some authors observed that the activity of this enzyme can be increased by the addition of some elicitors, such as octadecanoids, methyl jasmonate, and coronatine, resulting in enhanced tocopherol content [42].

From a general perspective, the elicitors used in our work stimulated plant defense mechanisms by accumulating secondary metabolites and phytoalexins that may allow tomato plants to rapidly react against biotic and abiotic stresses. These changes in plant secondary metabolism following elicitor application were elicitor dependent. PAs, SA, and BTH triggered plant responses different from chitosan that could be linked to the perception of the elicitor by the plants and the phytohormone-mediated signaling cascade. SA is an essential signal molecule for inducing SAR while ISR is commonly regulated by JA and ethylene [31]. Although ISR is typically activated upon colonization of plant roots by beneficial microorganisms, it is also known that molecules such as chitosan that are derived from the degradation of cells or cell walls might be involved in eliciting the systemic signal and ISR [43]. Therefore, our findings suggest that PAs, SA, and BTH, as endogenous elicitors, might trigger a SAR-related response while chitosan might induce a ISR-like response.

## 4. Materials and Methods

### 4.1. Plant Material, Growth Conditions, and Treatments with Elicitors

In the middle of April 2019, organically grown tomato plants (*S. lycopersicum*, cultivar Heinz 1301) were supplied by a local nursery at the three true leaves stage to be transplanted and grown at the experimental facility at Università Cattolica del Sacro Cuore, Piacenza, Italy. Plants were transplanted in 40 cm pots using a commercial loam substrate (Compo star, pH = 6.1 in water, EC 0.6 dS/m, density 375 kg/m^3^ and porosity 80% *v*/*v*). The plants grown under controlled conditions, with temperature of 18–25 °C and photoperiod of 16 h light/8 h darkness. The pots were watered with 2 L of water every three to four days. A total of 2 L of basic nutrient solution (13 mmol/L NO_3_-N, 1 mmol/L NH_4_-N, 1.75 mmol/L S, 1.5 mmol/L P, 5 mmol/L K, 4.5 mmol/L Ca, 2 mmol/L Mg, 1 mmol/L Na, 1 mmol/L Cl, 20 µmol/L Fe, 9 µmol/L Mn, 0.3 µmol/L Cu, 1.6 µmol/L Zn, 20 µmol/L B, and 0.3 µmol/L Mo) with an electrical conductivity of 2.0 dS/m was applied at 15 days after transplantation [44].

Six different treatments were prepared: a negative control (no treatments), a second negative control treated with 1 M acetic acid (the reference for chitosan, since chitosan was solubilized in acetic acid), and four molecular elicitors, namely 2,1,3-benzothiadiazole (BTH, a positive control), chitosan, SA, and a PA mix (Put, Spd, and Spm). Each treatment included five different pots of three plants each, and applications were done at the growth stage of nine true leaves unfolded on the main shoot. Treatments were performed by a manual sprayer; for each treatment, a volume of 6 mL was applied to each pot for an amount of about 2 mL for each plant. For spraying, the following solutions were prepared in double-distilled water: 1 mM of 2,1,3-Benzothiadiazole (BTH), (Sigma-Aldrich from Merck, Darmstadt, Germania, 98% purity) 10 mg/mL of chitosan from shrimp shells, technical grade (Sigma-Aldrich) in 1 M acetic acid (Sigma- Aldrich from Merck Darmstadt, Germania, ≥99% purity), 0.01 mM SA (Sigma-Aldrich, ≥99% or purity), and 0.1 mM of a polyamine mixture (Spm, ≥97%; Spd, ≥99%, and Put, ≥98%, Sigma-Aldrich from Merck, Darmstadt, Germania in the ratio of 1:1:1, *v*/*v*).

At 15 days after treatment, tomato plants were manually harvested, and leaves collected. Two distinct samplings, one for biomass and one for metabolomics, were carried out; in both cases, five leaves were harvested from each plant and pooled. A pool of leaves was used for fresh/dry weight determination (after drying at 80 °C until constant weight). The second portion of leaves was immediately quenched in liquid nitrogen and stored at −18 °C for metabolomics analysis.

### 4.2. Plant Biomass and Metabolomics Analysis by UHPLC/QTOF-MS

For metabolomics, leaves were homogenized in liquid nitrogen by pestle and mortar and then extracted in triplicate in 80% aqueous methanol acidified with 0.1% (*v*/*v*) formic acid, as previously reported [45]. Thereafter, the extracts were centrifuged at 7168× *g* for 10 min at 4 °C, and the supernatants were filtered in amber vials for analysis. Untargeted metabolomics were carried out by UHPLC liquid chromatography with quadrupole-time-of-flight mass spectrometry (UHPLC/QTOF-MS) (Agilent Technologies, Santa Clara, CA, USA) at Universitá Cattolica del Sacro Cuore (Piacenza, Italy), as previously reported [46]. Chromatographic separation used a binary linear gradient of water and acetonitrile (6–94% organic in 33 min) and a PFP column (2.0 × 100 mm, 3 µm—Agilent technologies, Santa Clara, CA, USA) with a flow rate 200 µLmin^−^^1^. For high-resolution mass spectrometry, the QTOF operated in SCAN mode (positive polarity, 100–1200 *m*/*z* range) and in extended dynamic range mode [47].

The software, Profinder B.07 (from Agilent Technologies), was used for deconvolution, mass and retention time alignment, and filtering (mass accuracy <5 ppm). Compound annotation used the whole isotopic pattern (monoisotopic mass, isotope spacing, and isotopes ratio) and the database, PlantCyc 12.6 (Plant Metabolic Network, http://www.plantcyc.org; accessed on 20 November 2021), leading to a level 2 of COSMOS confidence in annotation [48].

### 4.3. Statistical Analysis

Biomass was analyzed by one-way ANOVA (*p* < 0.05, Duncan’s post-hoc test) by means of the software, IBM PASW Statistics 26.0 (SPSS Inc., Chicago, IL, USA). Thereafter, the software, Mass Profiler Professional 12.6 (Agilent Technologies), was used for chemometric interpretation of metabolomics data according to Mimmo and co-workers [49]. In brief, post-acquisition normalization at the 75th percentile and baselining to the median of each compound were done; thereafter, unsupervised patterns were explored through a fold-change-based hierarchical cluster analysis using Euclidean distance. The dataset was then exported into SIMCA 16 (Umetrics, Malmo, Sweden) to be elaborated via OPLS-DA supervised modeling. Outliers were investigated by Hotelling’s T2 (95% and 99% confidence limits), validation was achieved by CV-ANOVA (*p* < 0.01), and overfitting excluded by permutation testing (*N* = 100). Thereafter, fitness parameters (goodness-of-fit, R^2^Y, and goodness-of-prediction, Q^2^Y), as well as VIP discriminant compounds (VIP score > 1.3) were recorded. Finally, ANOVA (*p* < 0.01, Bonferroni multiple testing correction) and fold-change (FC) analysis (FC ≥ 7) were combined, and differential compounds were imported into the Omic Viewer Pathway Tool of PlantCyc (Stanford, CA, USA) software [50] for biochemical interpretations.

## 5. Conclusions

The application of the four elicitors has been shown to trigger a broad metabolic reprogramming in tomato, mainly relating to secondary metabolism. Such shaping of plant biochemical processes included stress-related compounds, such as phenylpropanoids and other phytoalexins, membrane lipids, and alkaloids. The complex crosstalk of plant phytohormones emerged as a pivotal process underlying the modulation of plant secondary metabolism. Interestingly, elicitor-specific metabolomics signatures could be observed, in turn suggesting specific rather than generalized effects of elicitors.

These results pave the way towards the possible use of elicitors as a sustainable tool in plant science and agriculture, by increasing crops resilience to biotic and abiotic stressors. Surely dedicated experiments (possibly including different stresses) are worthy to be carried out in this direction.

## Figures and Tables

**Table 1:** Effect of the elicitors on leaf fresh weight and leaf dry weight after 15 days of treatment. Statistically homogenous groups are identified by letters (Duncan’s post-hoc, *p* ≤ 0.05).

Treatment	Fresh Weight (FW, g)	Dry Weight (DW, g)
Control	2.43 ± 0.25 a	0.49 ± 0.09 ab
Chitosan	2.15 ± 0.05 ab	0.39 ± 0.02 a
BTH	2.57 ± 0.25 b	0.50 ± 0.04 ab
Acetic acid	2.57 ± 0.15 b	0.49 ± 0.15 ab
SA	2.58 ± 0.04 b	0.49 ± 0.04 ab
PAs	3.41 ± 0.04 c	0.59 ± 0.04 b

## Data Availability

Data is provided as supplementary material.

## Supplementary Materials

The following are available online at https://www.mdpi.com/article/10.3390/plants11050678/s1, Figure S1. Effects of elicitors on shoot fresh (A) and dry (B) weight after 15 days of treatment. Statistically homogenous groups were identified by letters (Duncan’s post-hoc, *p* ≤ 0.05). Figure S2. List of the secondary metabolites mainly involved in tomato response to elicitor treatments; compounds are grouped in glucosinolates, alkaloids, phenylpropanoids, and terpenes, and individual fold-change values are plotted per each compound and each treatment within the corresponding biochemical class. Table S1. Dataset from untargeted metabolomics of tomato leaves after 15 days of treatment. Compounds are presented with individual intensities and with composite mass spectra. Table S2. Discriminant metabolites identified by the VIP analysis in tomato leaves after 15 days of treatment. Compounds were selected as discriminant by possessing a VIP score > 1.25. Table S3. Significant metabolites involved in plant response to elicitors and uploaded to the PlantCyc pathway Tool software derived from ANOVA (*p* < 0.01, Bonferroni multiple testing correction) and fold-change (FC) analysis (FC ≥ 7).
