# Supplementary material for: The Complex Metabolomics Crosstalk Triggered by Four Molecular Elicitors in Tomato
Source: Plants (Basel). 2022 Mar 1;11(5):678. doi: 10.3390/plants11050678 (PMC8912670; doi:10.3390/plants11050678)
Supplement: Supplementary file 1 [file plants-11-00678-s001.zip › Figure S2.pdf]

NITROGEN-CONTAINING SECONDARY METABOLITES

GLUCOSINOLATES

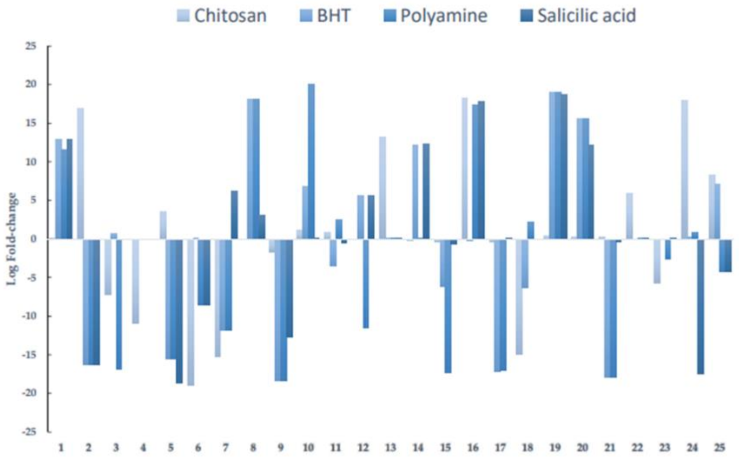

ALKALOIDS

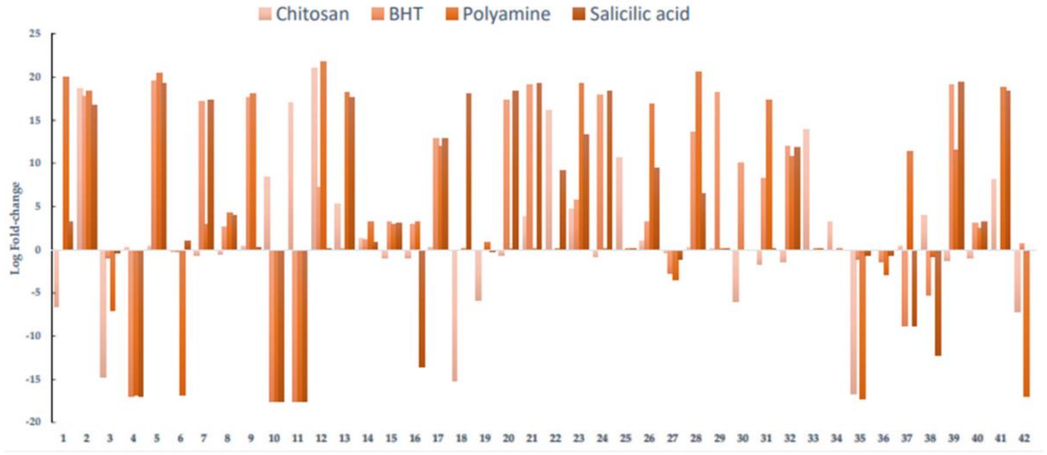

PHENYLPROPANOIDS

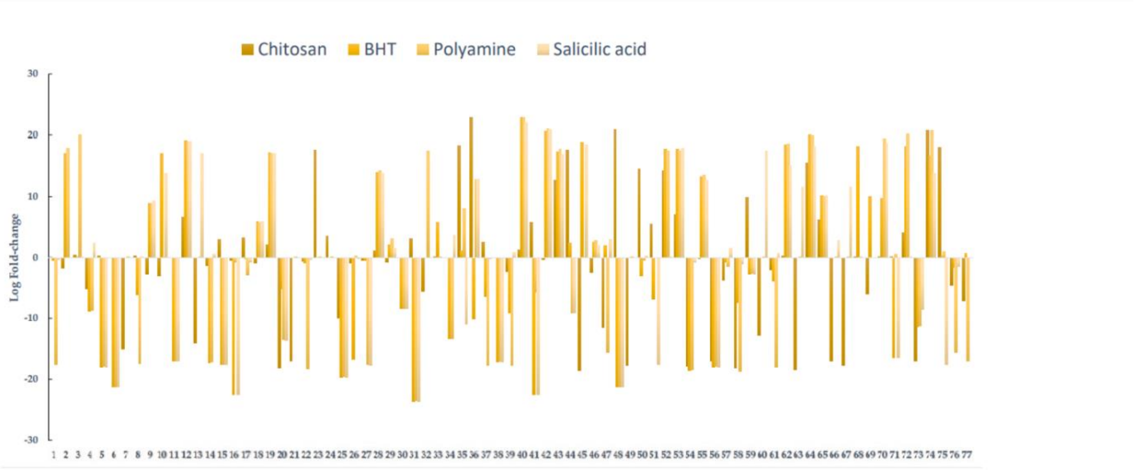

TERPENES

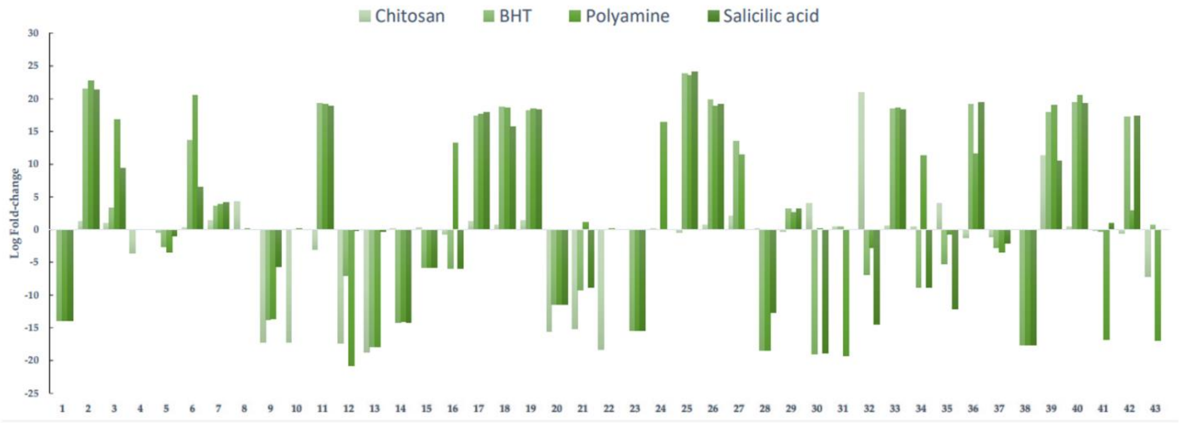

Figure S2. List of the secondary metabolites mainly involved in tomato response to elicitor treatments; com-pounds are grouped in glucosinolates, alkaloids, phenylpropanoids and terpenes, and individual fold-change values are plotted per each compound and each treatment, within the corresponding biochemical class. Nitrogen-containing secondary metabolites (glucosinolates and alkaloids), phenylpropanoids and terpenes and related compounds.

Legend Figure S2.

Number axis X	Glucosinolate related compound	Chitosan	BTH	PA	SA
1	(Z)-1-(L-cystein-S-yl)-N-hydroxy-2-phenylethan-1-imine	0.06	12.85	11.62	12.90
2	8-(methylsulfanyl)octyl-glucosinolate	16.91	-16.27	-16.22	-16.27
3	S-adenosyl-L-homocysteine (SAH)	-7.17	0.63	-16.93	0.01
4	4-methoxyglucobrassicin	-10.84	-0.07	0.01	-0.06
5	(E)-(indol-3-yl)acetaldehyde oxime	3.50	-15.52	-15.49	-18.59
6	(E)-1-(L-cysteinylglycin-S-yl)-N-hydroxy-2-(1H-indol-3-yl)ethan-1-imine	-18.94	0.08	-8.57	-8.50
7	6-(methylsulfanyl)hexyl-desulfoglucosinolate	-15.24	-11.83	-11.75	6.19
8	(E)-ω-(methylsulfanyl)heptyl-thiohydroximate	-0.08	18.14	18.13	3.07
9	(E)-1-(L-cysteinylglycin-S-yl)-N-hydroxy-ω-(methylsulfanyl)heptan-1-imine	-1.70	-18.38	-18.31	-12.75
10	3-[(5'-methylsulfanyl)pentyl]malate	1.12	6.80	20.02	0.04
11	L-hexahomomethionine	0.88	-3.53	2.44	-0.44
12	6-(methylsulfanyl)-2-oxohexanoate	-0.03	5.68	-11.52	5.52
13	N, N-dihydroxytrihomomethionine	13.23	0.06	0.14	0.10
14	L-trihomomethionine	-0.26	12.13	0.10	12.26
15	(E)-1-(L-cystein-S-yl)-N-hydroxy-ω-(methylsulfanyl)hexan-1-imine	-0.34	-6.10	-17.38	-0.61
16	(E)-1-(L-cysteinylglycin-S-yl)-N-hydroxy-ω-(methylsulfanyl)hexan-1-imine	18.22	-0.13	17.38	17.80
17	7-(methylsulfanyl)heptyl-glucosinolate	-0.31	-17.19	-17.01	0.13
18	(E)-ω-(methylsulfanyl)octyl-thiohydroximate	-14.88	-6.32	2.15	-0.07
19	(E)-1-(L-cysteinylglycin-S-yl)-N-hydroxy-ω-(methylsulfanyl)octan-1-imine	0.39	18.92	19.03	18.74
20	N,N-dihydroxy-L-dihomomethionine	0.30	15.52	15.60	12.09
21	4-(methylsulfanyl)butyl-desulfoglucosinolate	0.33	-17.96	-17.96	-0.33
22	(E)-1-(L-cysteinylglycin-S-yl)-N-hydroxy-ω-(methylsulfanyl)pentan-1-imine	5.84	0.02	0.10	0.04
23	N,N-dihydroxyhomomethionine	-5.69	-0.11	-2.59	0.15
24	adenosine 3',5'-bisphosphate (3',5'-ADP)	17.99	0.27	0.91	-17.49
25	(E)-1-(glutathion-S-yl)-N-hydroxy-ω-(methylsulfanyl)butan-1-imine	8.34	7.07	-4.17	-4.23

Number axis X	Alkaloid related compound	Chitosan	BTH	PA	SA
1	(S)-norreticuline	-6.6	0.02	19.99	3.17
2	cephaeline	18.68	17.7	18.35	16.72
3	deacetylisoipecoside	-14.67	-0.89	-7.03	-0.33
4	solanidine	0.21	-16.99	-16.85	-16.92
5	geraniol	0.42	19.48	20.47	19.28
6	loganin	-0.13	-0.19	-16.82	1.04
7	loganate	-0.57	17.16	2.88	17.31
8	lupanine	-0.5	2.56	4.23	4.02
9	13-hydroxylupanine	0.46	17.54	18	0.21
10	elwesine	8.36	-17.53	-17.53	-17.49
11	maritinamine	17.05	-17.61	-17.53	-17.6
12	11-hydroxyvittatine	21.08	7.23	21.78	0.04
13	4'-O-methylnorbelladine	5.36	0.07	18.21	17.57
14	lycorine	1.26	1.2	3.18	0.88
15	camptothecin	-0.95	3.26	2.89	3.05
16	(S)-stylopine	-0.89	2.86	3.28	-13.59
17	protopine	0.33	12.84	12	12.86
18	6-hydroxyprotopine	-15.24	0.02	0.1	18.04
19	10-hydroxydihydrosanguinarine	-5.76	-0.1	0.8	-0.16
20	sanguinarine	-0.59	17.35	0.1	18.32
21	(S)-nandinine	3.85	19.11	0.1	19.19
22	3-methylxanthine	16.12	0.02	0.1	9.23
23	chelerythrine	4.77	5.75	19.29	13.25
24	(S)-norcoclaurine	-0.84	17.97	0.1	18.33
25	codeinone	10.68	0.02	0.1	0.04
26	cholesterol	1.02	3.28	16.81	9.43
27	17-O-deacetylvindoline	-0.37	-2.68	-3.45	-1.01
28	(3R)-3-hydroxy-16-methoxy-2,3-dihydrotabersonine	0.23	13.6	20.57	6.48
29	(6R)-4a-hydroxy-tetrahydrobiopterin	0.14	18.18	0.09	0.04
30	(6R)-L-erythro-5,6,7,8-tetrahydrobiopterin	-5.99	10.03		-0.06
31	leucodopachrome	-1.65	8.26	17.25	0.04
32	cyclo-dopa 5-O-glucoside	-1.35	12.02	10.85	11.85
33	dopaxanthin	13.97	0.02	0.1	0.04
34	betanidin	3.26	-0.02	0.06	
35	2-descarboxy-betanidin	-16.65	-1.06	-17.24	-0.57
36	2-descarboxy-cyclo-dopa	-0.02	-1.38	-2.84	-0.67
37	16-epivellosimine	0.39	-8.74	11.34	-8.74
38	sarpagine	4	-5.19	-0.74	-12.16
39	10-deoxysarpagine	-1.2	19.16	11.52	19.42
40	3-O-acetyl-4'-O-demethylpapaveroxine	-0.91	3.01	2.51	3.17
41	(S)-reticuline	8.19	-0.03	18.75	18.31
42	S-adenosyl-L-homocysteine (SAH)	-7.17	0.63	-16.93	0.01

Number axis X	Phenylpropanoid related compound	Chitosan	BTH	PA	SA
1	7,4'-dimethylquercetin	-0.08	-0.44	-17.6	-0.36
2	afrormosin-7-O-glucoside-6''-O-malonate	-1.69	17.05	17.88	0.04
3	sulfuretin 6-glucoside	0.43	0.1	19.98	0.1
4	baicalin	-5.05	-8.73	-8.66	2.37
5	isovitexin-7-O-glucosyl-2''O-rhamnoside	0.18	-17.94	-17.86	-17.92
6	isovitexin 7-O-glucoside	-21.28	-21.28	-21.28	
7	isovitexin 2''-O-rhamnoside	-15.04	0.02	0.1	0.04
8	(+)-sesamolin	0.29	-6.04	-17.46	0.14
9	(+)-sesamin	-2.68	8.94	8.91	9.28
10	(+)-sesaminol 2-O-β-D-gentiobioside	-3.02	17.12	0.1	13.84
11	scoparone	0.05	-16.95	-16.93	-16.96
12	pinosylvin monomethylether	6.62	19.18	18.9	19.01
13	hypericin	-14.07	0.02	0.1	17.07
14	protohypericin	-1.33	-17.24	-17.15	0.57
15	2-O-acetyl-3-O-trans-coutarate	2.97	-17.6	-17.52	-17.58
16	cyanidin 3-O-(6-O-β-D-glucosyl-2-O-β-D-xylosyl-β-D-galactoside)	-0.4	-22.43	-0.73	-22.43
17	(-)-medicarpin-3-O-glucoside	3.26	-0.2	-2.81	-0.71
18	butyl propanoate	-0.93	5.95	5.76	5.95
19	quercetin 3,4'-O-diglucoside	2.11	17.23	17.14	17
20	glyceollin III	-18.11	-5.11	-13.4	-13.53
21	kaempferol 3-O-β-D-glucoside	-16.93	0.02	0.1	0.04
22	(S)-malate	-0.55	-0.86	-18.32	-0.37
23	myricetin 3-O-gentiobioside	17.61	0.02	0.1	0.04
24	myricetin 3-O-β-D-glucoside	3.51	-0.02	0.06	
25	(+)-pinoresinol	-9.93	-19.63	-19.55	-19.61
26	7,2,2'-trihydroxy-4',5'-methylenedioxyisoflav-3-ene	-0.89	-16.66	0.2	-0.15
27	pilosin	-0.5	-0.44	-17.6	-17.63
28	phlorizin	1.05	13.96	14.18	13.86
29	(Z)-6'-hydroxyferulate	-0.72	2.1	3.04	1.55
30	alizarin		-8.41	-8.41	-8.41
31	cis-coumarinic acid-β-D-glucoside	3.08	-23.63	-23.55	-23.61
32	syringetin	-5.51	0.02	17.55	0.04
33	3,7-dimethylmyricetin	0.14	5.72	0.06	
34	desmethylxanthohumol	-0.07	-13.27	-13.29	3.59
35	patuletin	18.26	1.15	8.08	-10.87
36	methylcinnamate	22.91	-10.04	12.86	12.88
37	(-)-epiafzelechin	2.48	-6.36	-17.78	-0.14
38	5-hydroxyferulate	0.01	-17.13	-17.06	-17.12
39	peonidin 3-O-glucoside	-2.35	-9.1	-17.66	0.88
40	cyanidin 3-O-β-D-caffeoylglucoside	1.21	22.82	22.91	22.08
41	cyanidin 3-O-β-D-(caffeoyl)-sambubioside	5.79	-22.55	-5.69	-22.54

42	β-D-apiofuranosyl-(1->6)-D-glucose	-0.3	20.61	21.05	20.84
43	3',5'-di-C-glucosylphloretin	12.74	17.28	17.75	16.88
44	phloretin	17.67	2.39	-9.02	-9.09
45	esculin	-18.51	18.94	0.17	18.46
46	3,7,3',4'-tetramethylquercetin 2'-O-β-D-glucoside	-2.39	2.53	2.85	1.89
47	psoralen	-11.5	2	-15.49	2.99
48	pelargonidin 3,7-di-O-β-D-glucoside	20.84	-21.26	-21.26	-21.26
49	apigenin 7-O-(6-malonyl-β-D-glucoside)	-17.63	0.02	0.1	0.04
50	scopolin	14.53	-3.06	-0.38	0.3
51	wighteone	5.41	-6.79		-17.47
52	isowighteone	14.24	17.77	17.5	0.12
53	lupiwighteone	7.03	17.77	17.5	17.92
54	delphinidin 3-O-rutinoside-7-O-glucoside	-17.79	-18.46	-18.38	-0.81
55	1-O-malyl-β-D-glucose	-0.15	13.2	13.55	12.66
56	justicidin B	-17.07	-17.94	-17.86	-17.98
57	geranylhydroquinone	-3.76	-0.8	-1.47	1.46
58	shikonin	-18.1	-7.35	-18.72	-0.94
59	(2S)-pinocembrin	9.79	-2.7	-2.62	-2.68
60	cyanidin 3-O-(3'',6''-O-dimalonyl-β-glucopyranoside)	-12.81	0.02	0.1	17.53
61	kaempferide 3-O-glucoside	-2.03	-3.84	-17.97	0.7
62	6-methoxymellein	0.33	18.52	18.64	15.12
63	hesperitin-7-O-β-D-glucoside	-18.43	0.02	0.1	11.53
64	2'-hydroxypseudobaptigenin	15.47	20.1	19.93	18.14
65	isoliquiritigenin	6.23	10.12	10.04	10.19
66	calycosin 7-O-glucoside	-17.05	0.02	0.1	2.75
67	1-O,6-O-digalloyl-β-D-glucose	-17.75	0.02	0.1	11.49
68	(6R)-4a-hydroxy-tetrahydrobiopterin	0.14	18.18	0.09	0.04
69	(6R)-L-erythro-5,6,7,8-tetrahydrobiopterin	-5.99	10.03		-0.06
70	(R)-3-(3,4-dihydroxyphenyl)lactate	0.12	9.68	19.53	18.79
71	luteolin 7-O-β-D-glucoside	0.1	-16.46	0.53	-16.45
72	rosmarinate	4.11	18.21	20.2	0.11
73	(-)-maackiain-3-O-glucoside	-17.05	-11.26	-11.18	-8.52
74	bisdemalonylsalvianin	20.75	16.63	20.74	13.9
75	adenosine 3',5'-bisphosphate (3',5'-ADP)	17.99	0.27	0.91	-17.49
76	3,6,7,3',4'-pentamethylquercetagetin	-4.6	-1.56	-15.5	-1.48
77	S-adenosyl-L-homocysteine (SAH)	-7.17	0.63	-16.93	0.01

Number axis X	Terpenes and related compound	Chitosan	BTH	PA	SA
1	ginsenoside Ro		-13.96	-13.96	-13.96
2	steviolmonoside	1.24	21.46	22.77	21.34
3	cholesterol	1.02	3.28	16.81	9.43
4	(R)-5-phosphomevalonate	-3.61	-0.03	0.05	-0.01

5	17-O-deacetylvindoline	-0.37	-2.68	-3.45	-1.01
6	(3R)-3-hydroxy-16-methoxy-2,3-dihydrotabersonine	0.23	13.6	20.57	6.48
7	heliocide B1	1.32	3.56	3.81	4.13
8	hemigossypolone-6-methyl ether	4.31	0.07	0.12	0.07
9	bis(β-D-glucosyl) crocetin	-17.24	-13.83	-13.7	-5.64
10	β-D-gentiobiosyl crocetin	-17.14	0.01	0.08	0.02
11	2-dehydrolubimin	-2.97	19.25	19.08	18.88
12	solavetivone	-17.34	-7	-20.8	-0.12
13	heliespirone B	-18.64	-17.81	-17.83	-0.27
14	crocetin	0.14	-14.14	-14.06	-14.13
15	2-cis,4-trans-xanthoxin	0.24	-5.82	-5.73	-5.78
16	(2E,6E)-farnesyl phosphate	-0.74	-5.84	13.21	-5.84
17	soyasapogenol B	1.28	17.35	17.64	17.84
18	16-alpha-hydroxygypsogenate	0.64	18.76	18.54	15.69
19	(+)-cis-abscisic aldehyde	1.39	18.24	18.42	18.39
20	trans-abscisic alcohol	-15.5	-11.42	-11.42	-11.42
21	tetrahydrogeranylgeranyl diphosphate	-15.2	-9.27	1.11	-8.86
22	all-trans phytofluene	-18.25	0.02	0.1	0.05
23	15-cis-phytoene		-15.37	-15.37	-15.37
24	2-phospho-4-(cytidine 5'-diphospho)-2-C-methyl-D-erythritol	0.14	0.02	16.4	0.04
25	4'-hydroxyadonixanthin	-0.48	23.77	23.56	24.1
26	gibberellin A17	0.66	19.82	18.9	19.08
27	gibberellin A5	2.12	13.46	11.41	0.04
28	oleanolate 3-beta-D-glucuronoside-(3,1)-galactoside	0.14	-18.45	-18.38	-12.68
29	geranial	-0.26	3.15	2.63	3.19
30	ent-kaurenol	4.06	-18.9	0.09	-18.88
31	gibberellin A12-aldehyde	0.43	0.39	-19.25	-0.01
32	ent-7α-hydroxykaur-16-en-19-oate	20.89	-6.86	-2.8	-14.39
33	dihydroartemisinate	0.53	18.45	18.55	18.28
34	16-epivellosimine	0.39	-8.74	11.34	-8.74
35	sarpagine	4	-5.19	-0.74	-12.16
36	10-deoxysarpagine	-1.2	19.16	11.52	19.42
37	monodeglucosyl des-acyl avenacin A	-1.08	-2.78	-3.46	-2.02
38	des-acyl avenacin A		-17.54	-17.54	-17.54
39	3-deoxycapsidiol	11.28	17.82	19	10.5
40	geraniol	0.42	19.48	20.47	19.28
41	loganin	-0.13	-0.19	-16.82	1.04
42	loganate	-0.57	17.16	2.88	17.31
43	S-adenosyl-L-homocysteine (SAH)	-7.17	0.63	-16.93	0.01
