# Supplementary material for: The Complex Metabolomics Crosstalk Triggered by Four Molecular Elicitors in Tomato
Source: Plants (Basel). 2022 Mar 1;11(5):678. doi: 10.3390/plants11050678 (PMC8912670; doi:10.3390/plants11050678)
Supplement: Supplementary file 1 [file plants-11-00678-s001.zip › Figure S1.pdf]

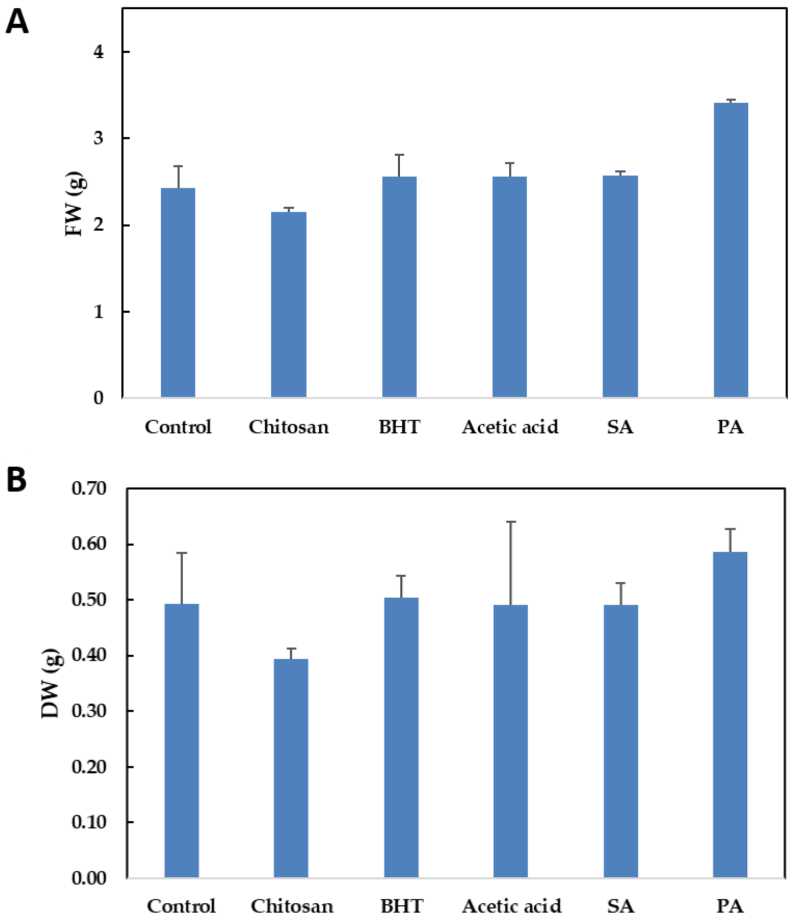

Figure S1. Effects of elicitors on shoot fresh (A) and dry (B) weight after 15 days of treatment. Statistically homogenous groups were identified by letters (Duncan's post-hoc, $P \leq 0.05$).
